# Supplementary material for: Platinum single-atom catalyst coupled with transition metal/metal oxide heterostructure for accelerating alkaline hydrogen evolution reaction
Source: Nat Commun. 2021 Jun 18;12:3783. doi: 10.1038/s41467-021-24079-8 (PMC8213696; doi:10.1038/s41467-021-24079-8)
Supplement: Supplementary file 1 — revised supporting information [file 41467_2021_24079_MOESM1_ESM.pdf]

Supporting Information

Platinum Single-Atom Catalyst Coupled with Transition Metal/metal Oxide Heterostructure for Accelerating Alkaline Hydrogen Evolution Reaction

Kai Ling Zhou,[a] Zelin Wang,[a] Chang Bao Han,*[a] Xiaoxing Ke,*[a] Changhao Wang,[a] Yuhong Jin,[a] Qianqian Zhang,[a] Jingbing Liu, [a] Hao Wang*[a] and Hui Yan[a]

[a.] Faculty of Materials and Manufacturing, Beijing University of Technology, Beijing 100124, P. R. China

E-mail: cbhan@bjut.edu; kexiaoxing@bjut.edu.cn.cn; haowang@bjut.edu.cn

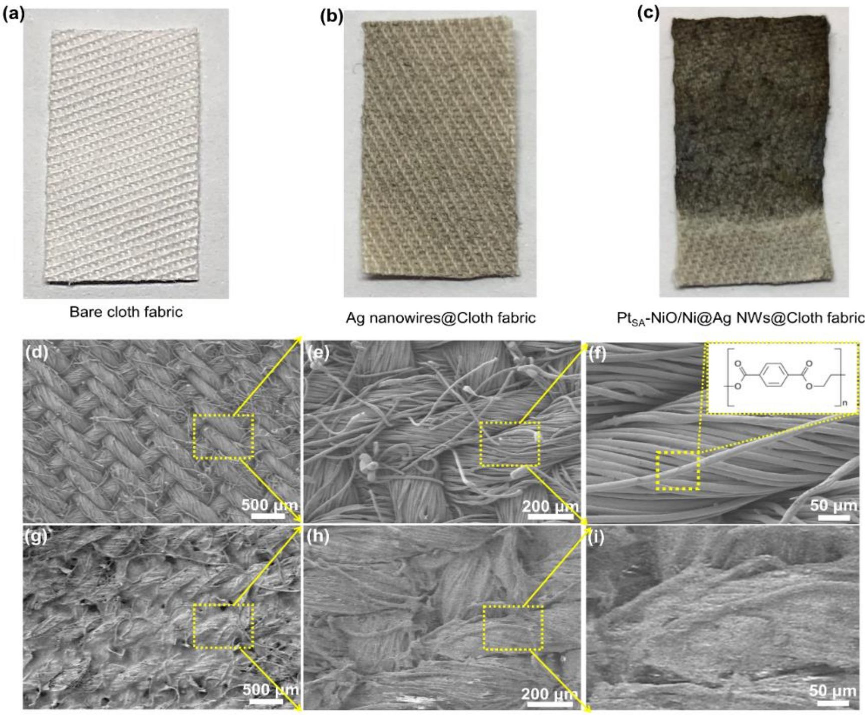

Figure S1. The digital images of (a) bare cloth fabric, (b) the Ag NWs loaded cloth fabric, and (c) the as-prepared Pt_{SA}-NiO/Ni@Ag NWs network on cloth fabric. The different-magnification SEM images of (d-f) the bare cloth fabric substrate and (g-i) the fabricated Pt_{SA}-NiO/Ni@Ag NWs on a cloth fabric substrate.

The cloth fabric was made from polyester fibers which have good elasticity, wrinkle resistance, shape retention, excellent wash-and-wear performance, and durability[1]. Besides, polyester fibers have good resistance to strong alkalies and acids at room temperature and belong to an insulator. The majority composition of polyester fibers is composed of terephthalic acid and ethylene glycol (PET) as shown in the insert in Figure S1f [2]. The favorable hydrogen release deriving from the interconnected pore structure and high stability originating from the good resistance to strong alkalies will contribute to cloth fabric being used as an excellent substrate for electrocatalytic hydrogen evolution in alkaline media.

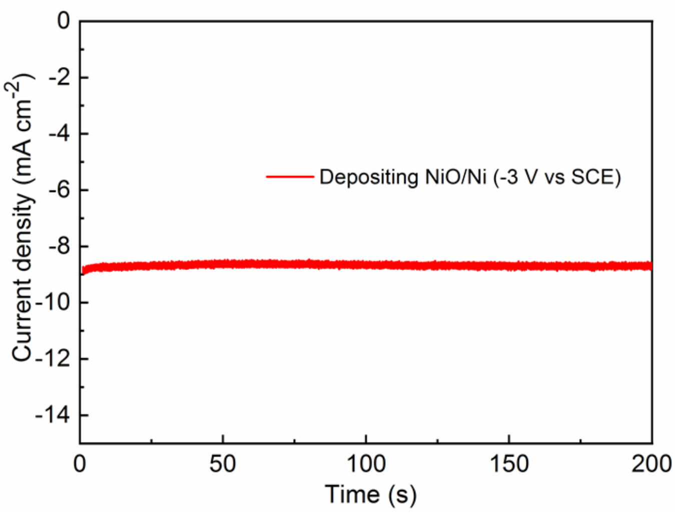

Figure S2. The electrochemical process with -3.0 V versus SCE for the deposition of NiO/Ni composite on the Ag NWs network.

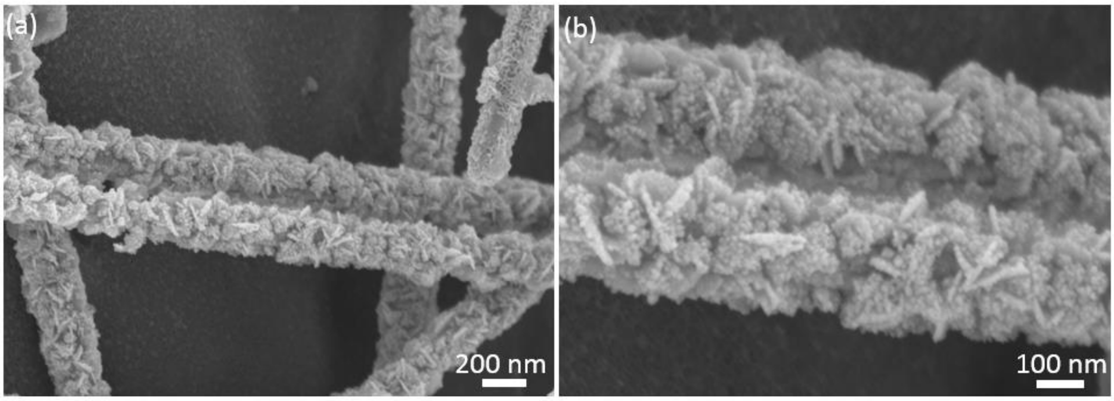

Figure S3. The SEM images of NiO/Ni on Ag NWs with different magnifications.

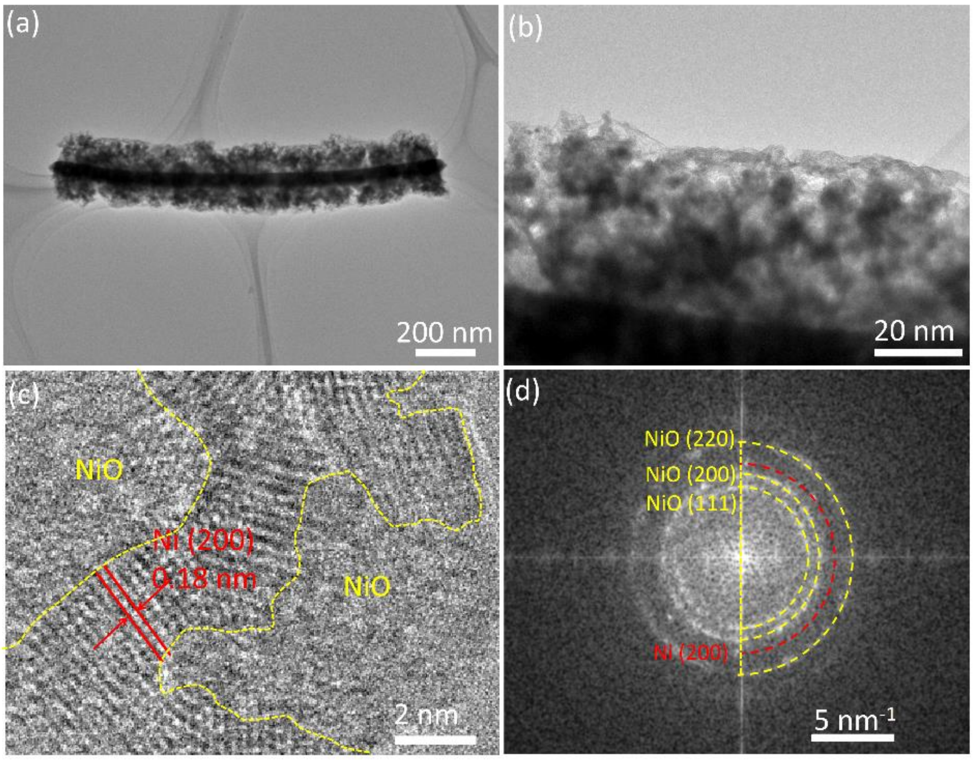

Figure S4. (a-b) Transmission electron microscopy (TEM) images, (c) high-resolution TEM (HRTEM), and (d) the corresponding fast Fourier transform (FFT) pattern of Ag NWs supported NiO/Ni.

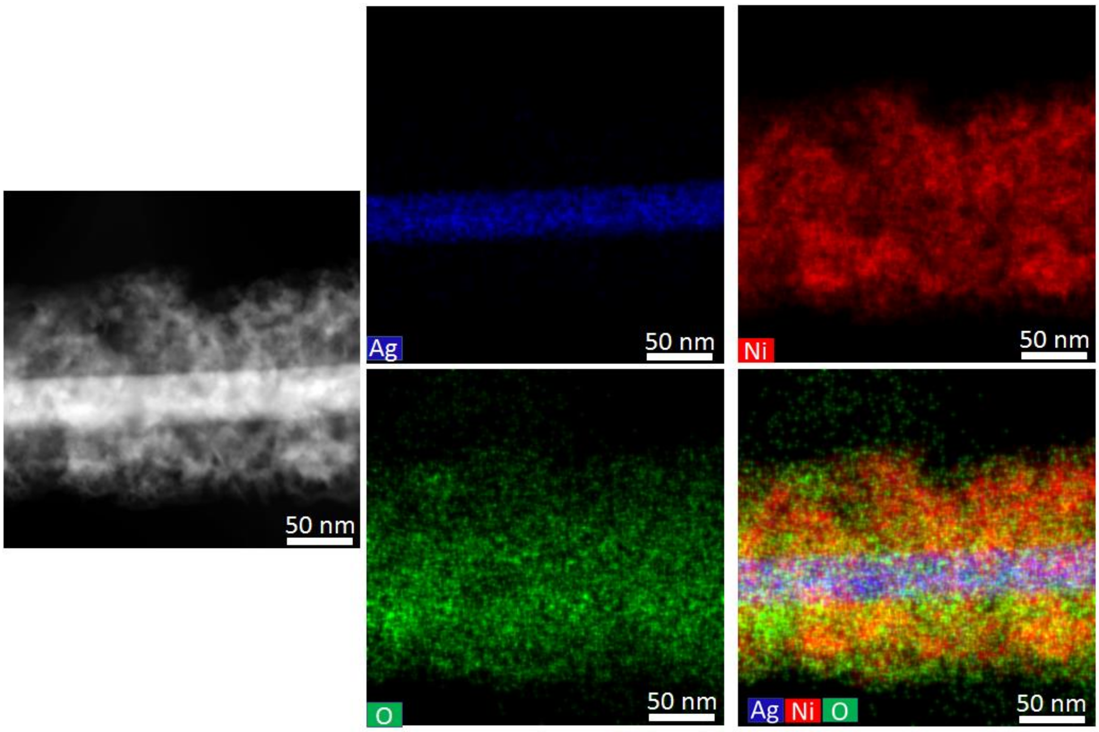

Figure S5. The elemental mapping of Ag, Ni, and O in Ag NWs supported NiO/Ni.

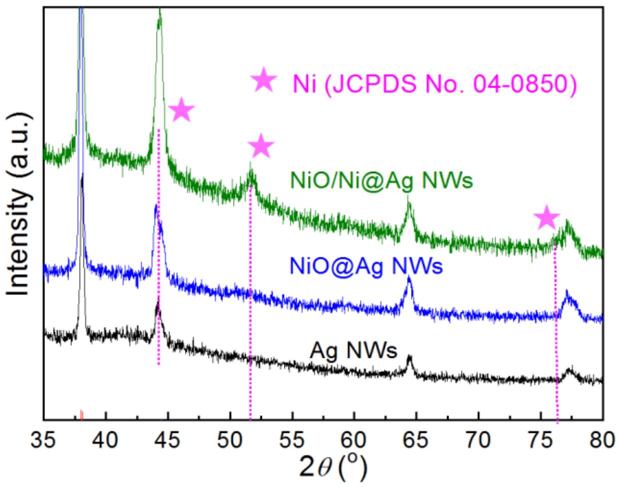

Figure S6. The X-ray diffraction (XRD) patterns of NiO/Ni and NiO on Ag NWs.

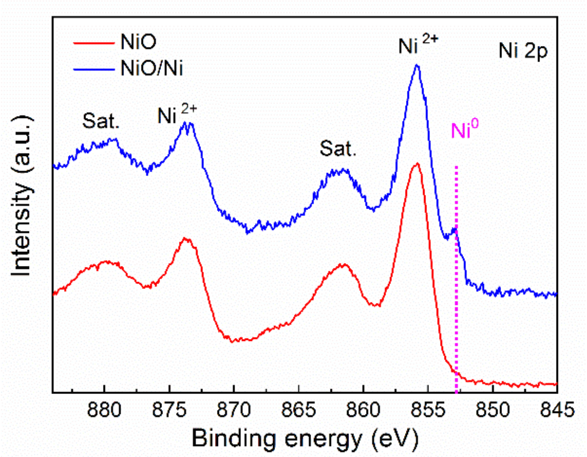

Figure S7. X-ray photoelectron spectroscopy spectra of NiO/Ni and NiO.

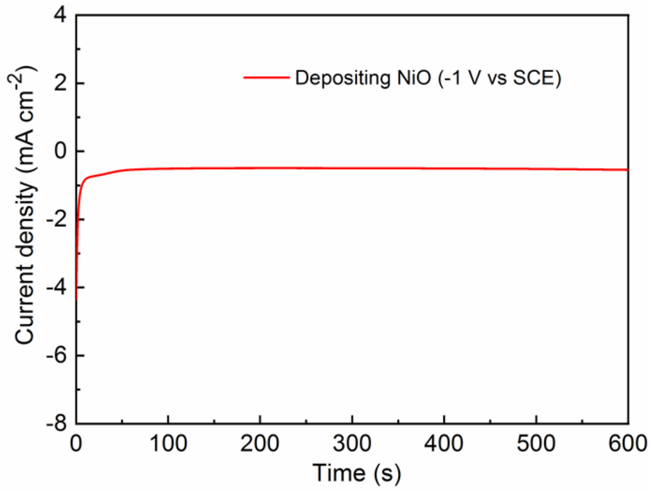

Figure S8. The electrochemical process with -1.0 V versus SCE for the deposition of NiO on the Ag NWs network.

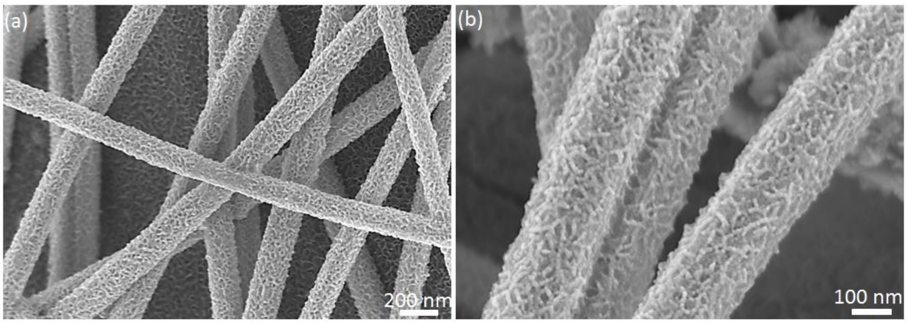

Figure S9. The SEM images of NiO on Ag NWs with different magnifications.

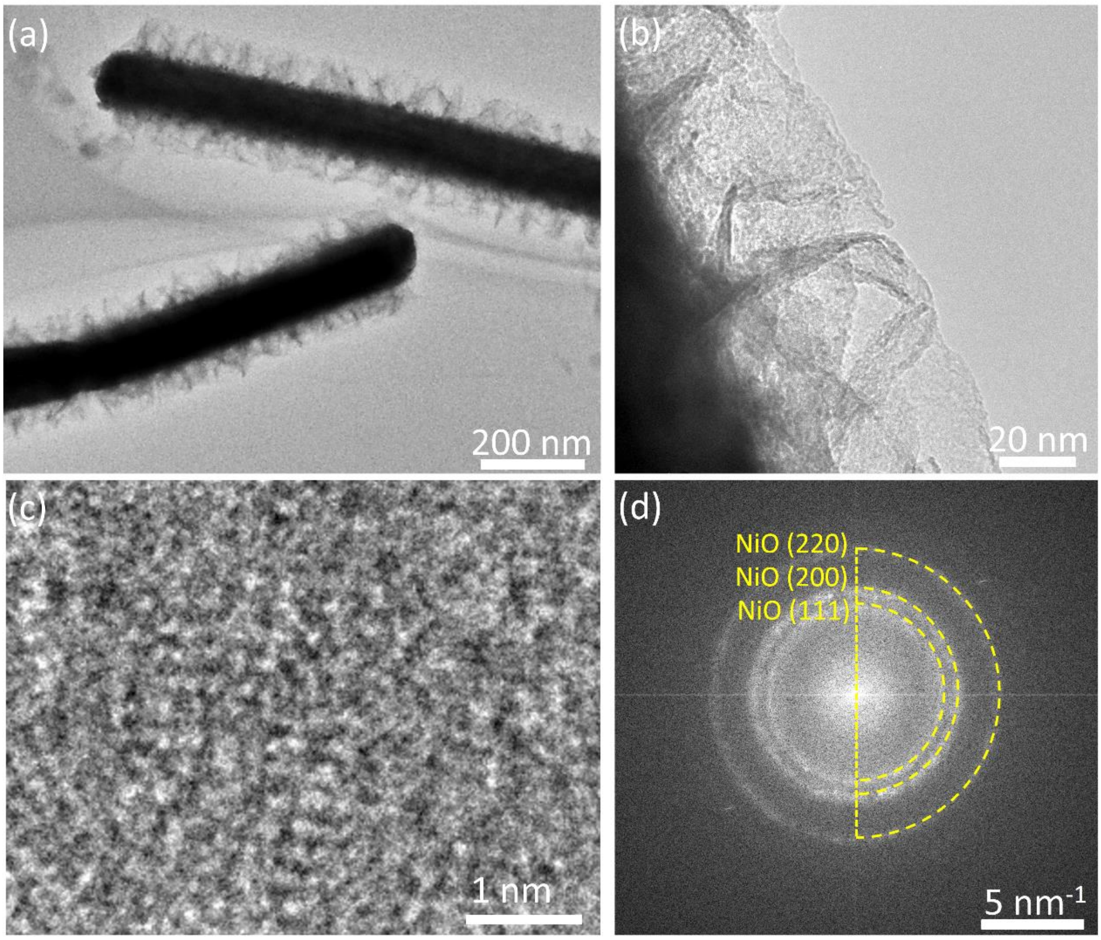

Figure S10. (a-b) TEM images, (c) HRTEM, and (d) FFT pattern of NiO on Ag NWs.

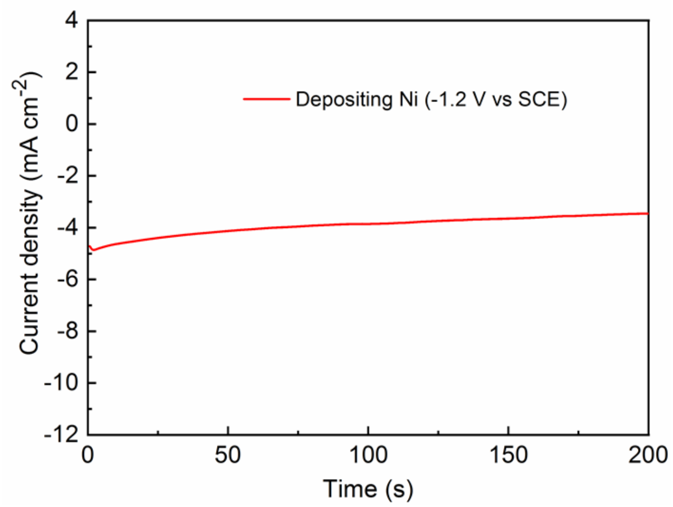

Figure S11. The electrochemical process with -1.2 V versus SCE for the deposition of metallic Ni on the Ag NWs network.

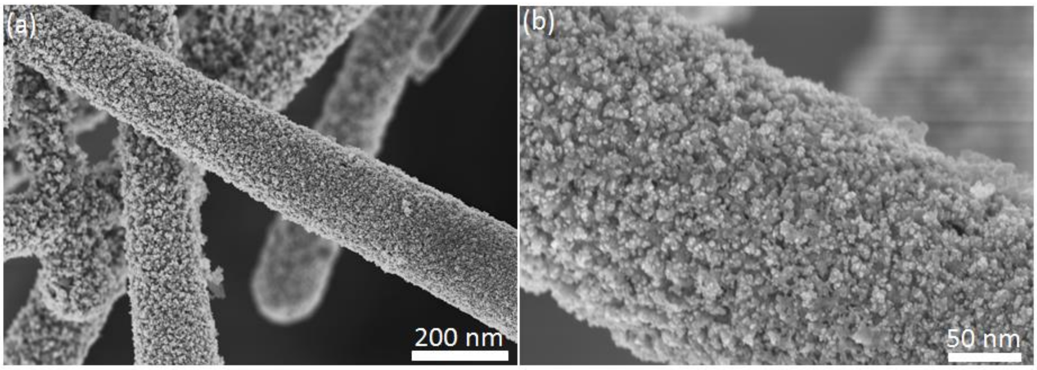

Figure S12. The SEM images of Ni on Ag NWs with different magnifications.

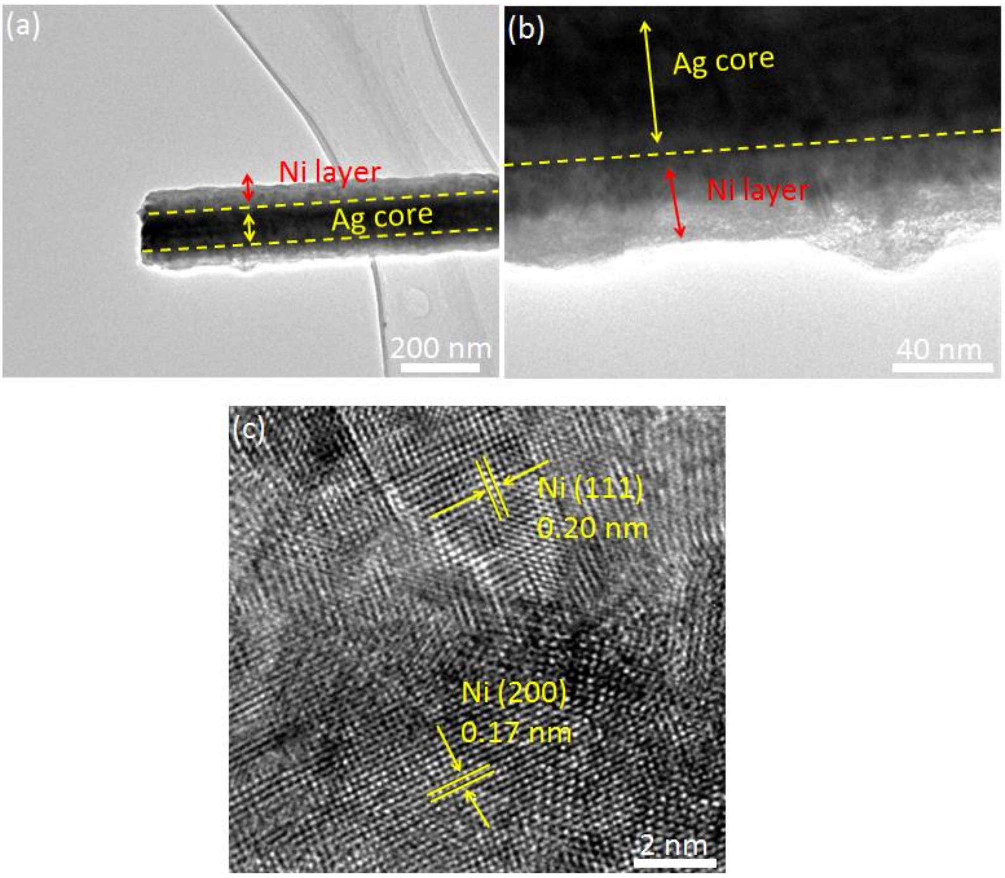

Figure S13. (a-b) TEM images and (c) HRTEM image of Ni.

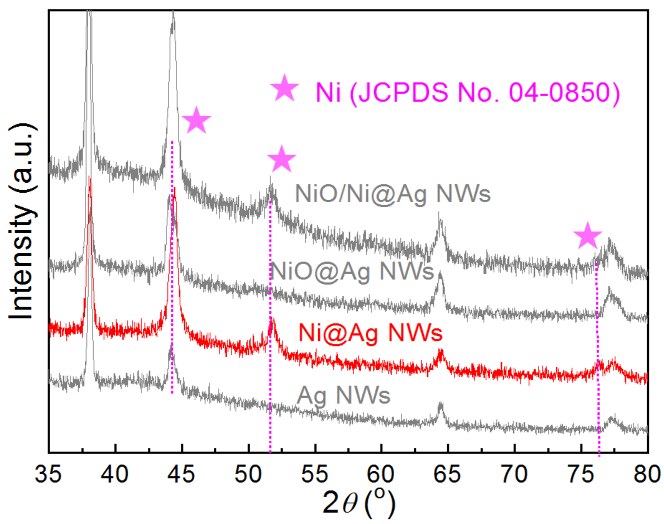

Figure S14. XRD patterns of Ag NWs supported Ni, and the XRD patterns of Ag NWs supported NiO/Ni and NiO are given as the comparison.

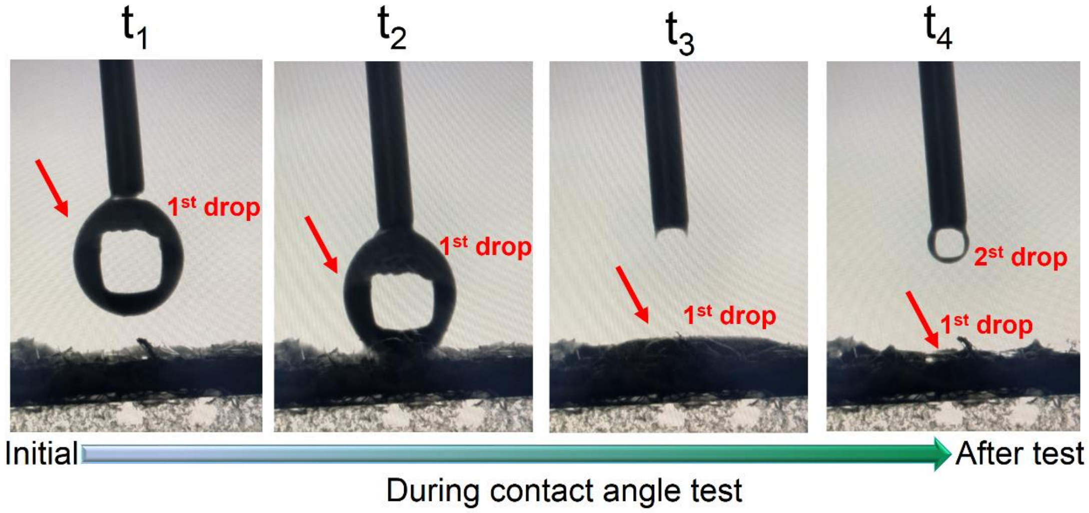

Figure S15. Contact angle measurement of the cloth fabric substrate supported Pt_{SA}-NiO/Ni@Ag NWs electrode.

The wettability of the cloth fabric supported catalyst was investigated by measuring the contact angle of the electrode. As shown in Figure S15, it was difficult to measure the contact angle of the electrode as the water was absorbed by the felt instantaneously, indicating the super hydrophilic nature. The excellent hydrophilicity will boost the electrolyte accessibility, accelerate the mass transfer, reduce the charge transfer resistance of the electrode and increase the durability of the electrode.[3]

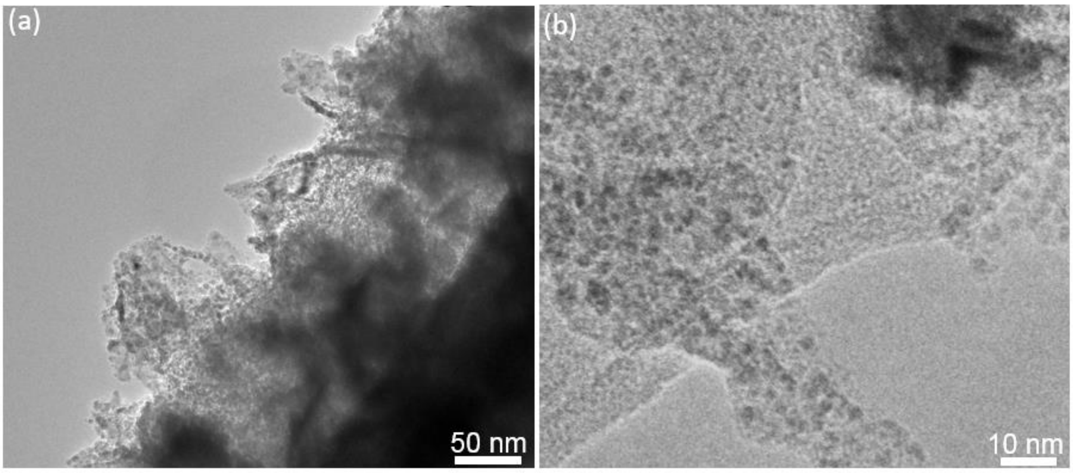

Figure S16. The TEM images of Pt_{SA}-NiO/Ni.

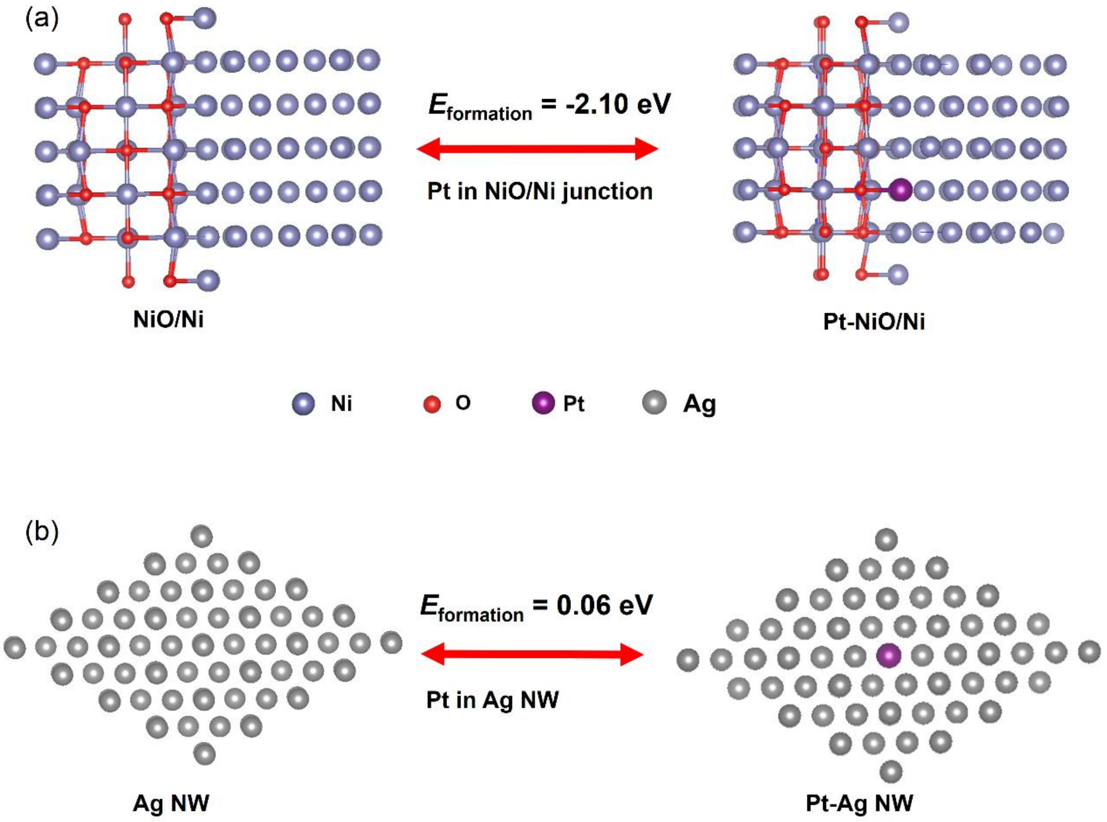

Figure S17. The optimized structures and the formation energy of Pt immobilized at the (a) NiO/Ni and (b) Ag NW.

According to the optimized crystal structures deriving from the DFT calculation (Figure S17), the formation energy of Pt immobilized at NiO/Ni is -2.10 eV, significantly lower than that of Pt immobilized at Ag NW (0.06 eV), suggesting the preference deposition of Pt atoms on NiO/Ni.

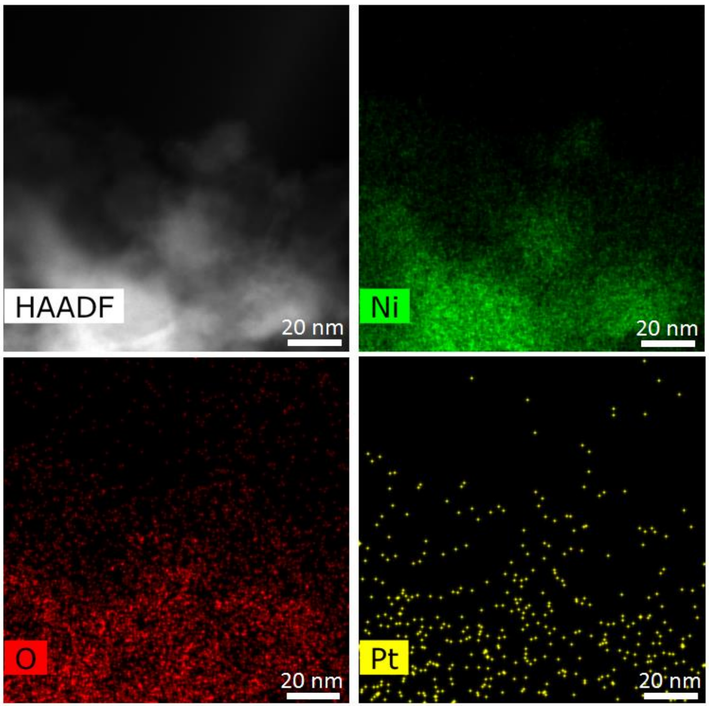

Figure S18. The elemental mapping of Ni, O, and Pt in Pt_{SA}-NiO/Ni.

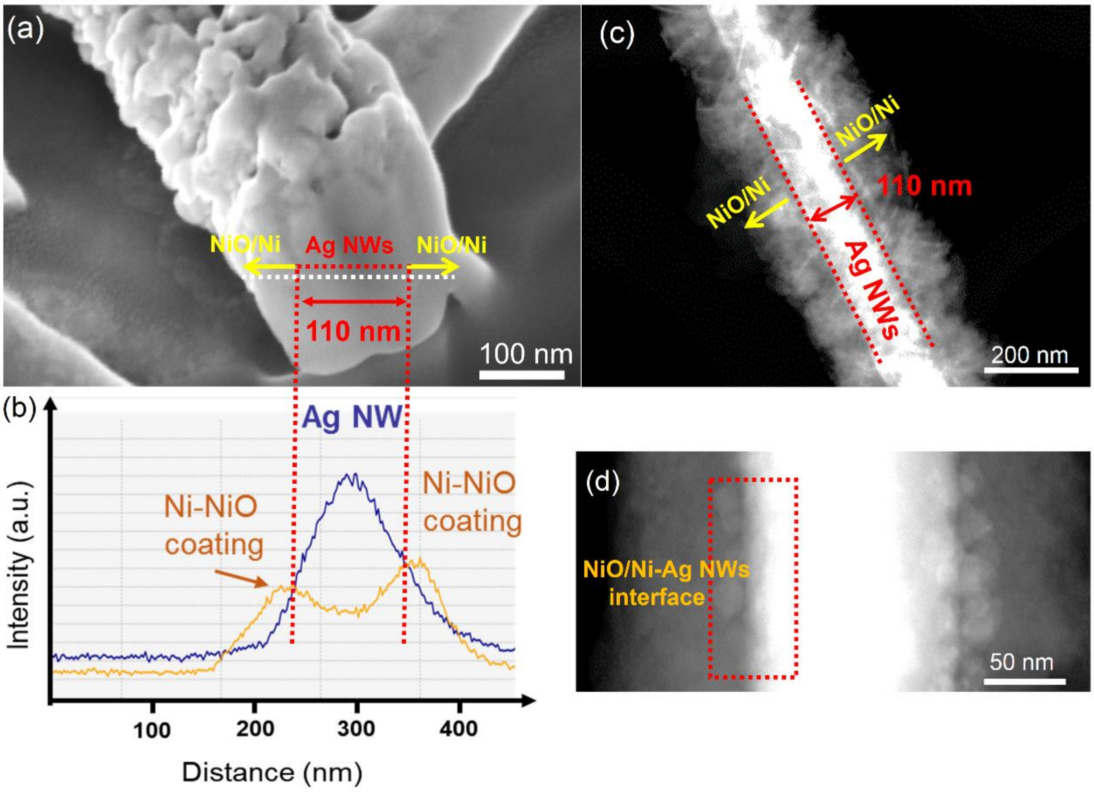

Figure S19. (a-b) The SEM image of the cross-section of Pt_{SA}-NiO/Ni@Ag NWs with the associated EDS line profile of Ag and Ni along the dotted white line. (c-d) The HAADF-STEM image of Pt_{SA}-NiO/Ni@Ag NWs.

The cross-sections of Pt_{SA}-NiO/Ni@Ag NWs are obtained by Focused Ion beam technique (FIB, FEI Helios Nanolab 600i FIB/SEM dual beam system equipped with Energy Dispersive System (EDS) detectors as shown in Figure S19a. Ag NWs are compactly coated by NiO/Ni layer, and no bare Ag surface could be observed. The EDS (Figure S19b) line profile of Ag and Ni elements along the cross-section of Pt_{SA}-NiO/Ni@Ag NWs further proves the compact NiO/Ni coating layer wrapping Ag NWs. HAADF-STEM (Figure S19c-d) image displays a seamless junction between NiO/Ni and Ag NW. The above results efficiently demonstrate the Ag NWs are compactly wrapped by the NiO/Ni layer, which could prevent the Ag from directly contacting and reacting with $PtCl_6^{2-}$ ions in KOH solution during the depositing process of Pt atoms.

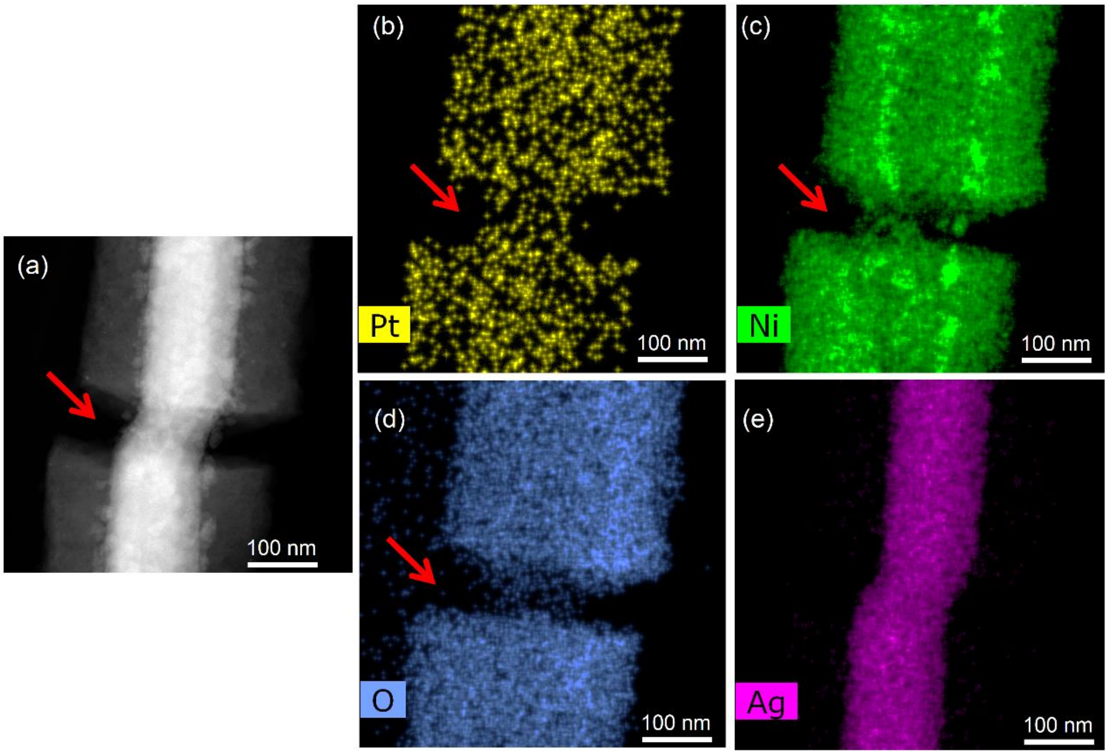

Figure S20. HAADF-STEM image and EDS mapping of Pt_{SA}-NiO/Ni@Ag NWs after the powerful ultrasonic treatment.

A core-shell structure of Pt_{SA}-NiO/Ni@Ag NWs after the powerful ultrasonic treatment was clearly shown in Figure S20. The “core” layer was composed of Ag element, and Ni, O, and Pt element share the same area located at the“shell” layer. The powerful ultrasonic treatment results in some damage and exfoliation of NiO/Ni shell layer as director of the red arrow in Figure S20. Interestingly, the evolution of Pt element distribution in the NiO/Ni shell area before and after ultrasonic treatment shows the same feature as that of Ni and O elements, proving that the Pt atoms mainly deposit on NiO/Ni.

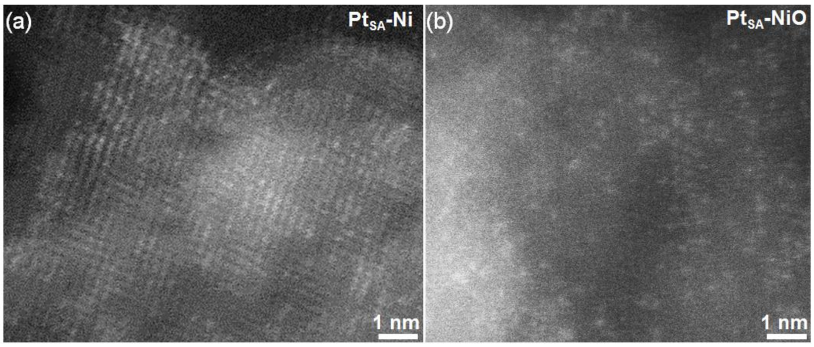

Figure S21. HAADF-STEM images of (a) Pt_{SA}-NiO and (b) Pt_{SA}-Ni, respectively.

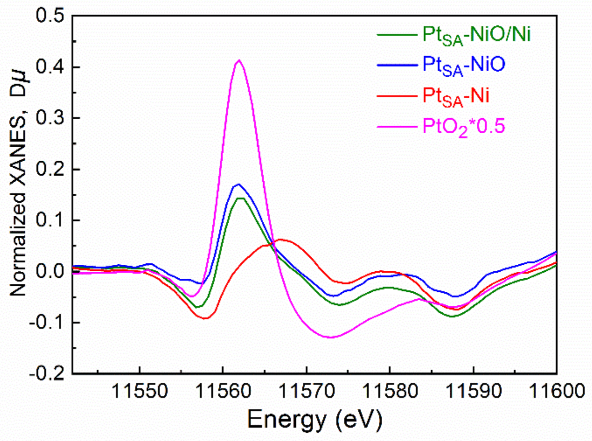

Figure S22. The normalized difference spectra for Pt L_3-edge XANES using Pt foil as reference. The oxidation states are fitted by integrating the area of the white-line peak from 11557 to 11574 eV.

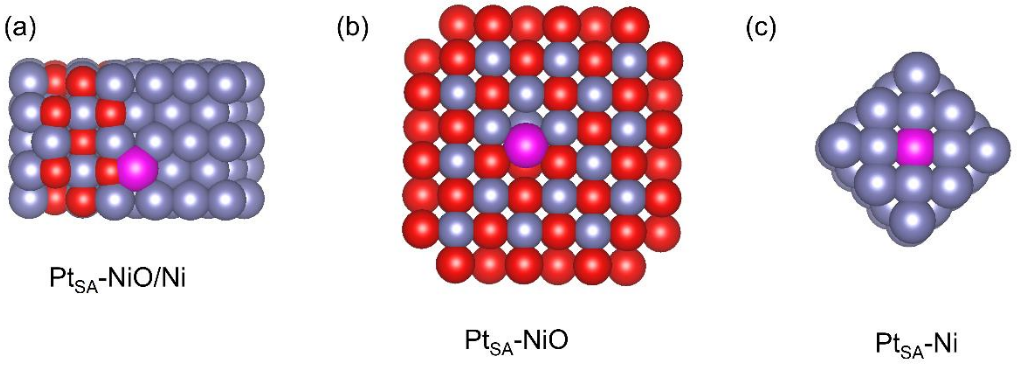

Figure S23. The DFT-optimized structure for (a) Pt_{SA}-NiO/Ni, (b) Pt_{SA}-NiO, and (c) Pt_{SA}-Ni catalysts, respectively, based on the EXAFS analysis.

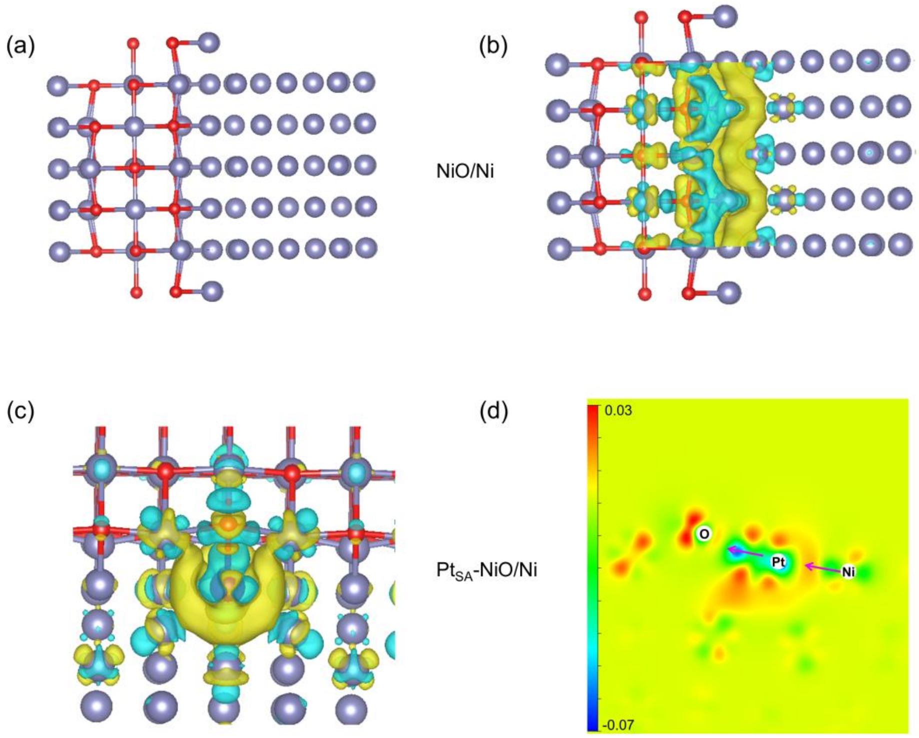

Figure S24. (a) Computational models of NiO/Ni heterostructure. (b) The localized electric field distribution of NiO/Ni heterostructure. (c) The localized electric field distribution of Pt atom at NiO/Ni phase junction. (d) 2D isosurface map of the cross-section consisting of Pt, Ni, and O atoms in Pt_{SA}-NiO/Ni with a unit of e A^{-3}.

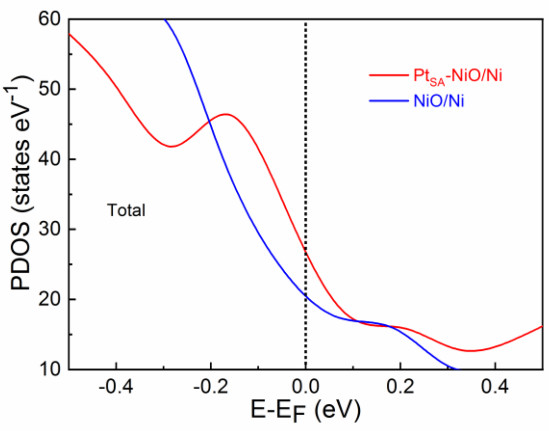

Figure S25. Highlighted total DOS of NiO/Ni and Pt_{SA}-NiO/Ni with aligned Fermi level.

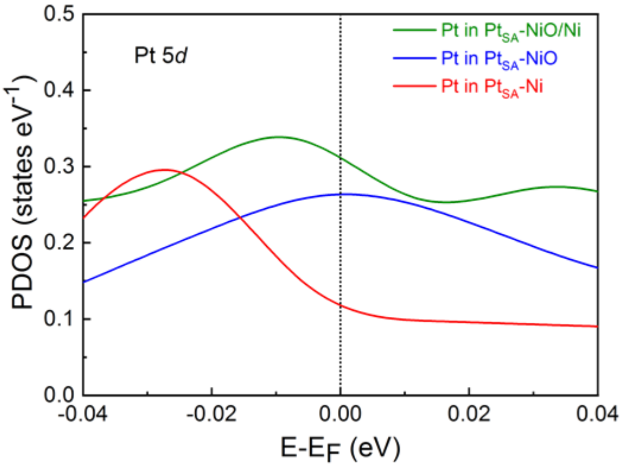

Figure S26. Highlighted Pt 5*d* band of Pt$_{SA}$-NiO, Pt$_{SA}$-Ni, and Pt$_{SA}$-NiO/Ni with aligned Fermi level.

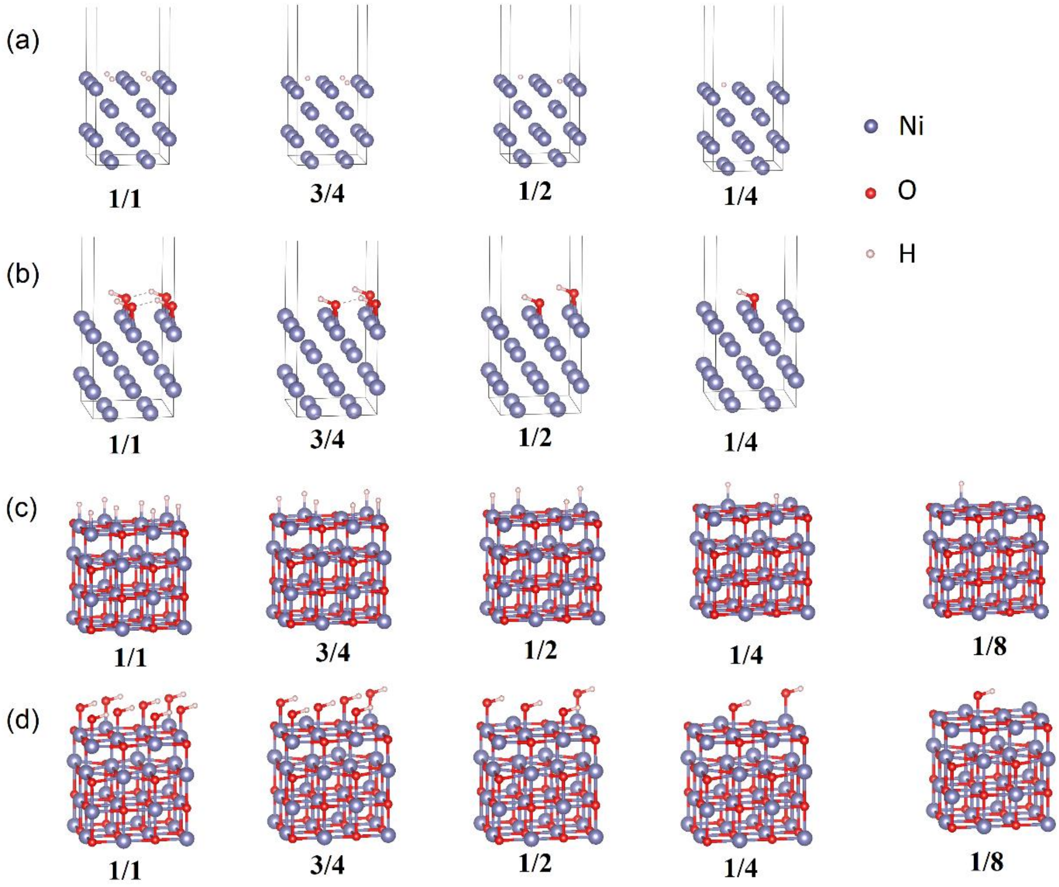

Figure S27. The H and OH adsorption energies of (a-b) Ni and (c-d) NiO with the different coverages of 1/1, 3/4, 1/2, 1/4, and 1/8.

The H and OH adsorption energies of Ni and NiO with the different coverages of 1/1, 3/4, 1/2, 1/4, and 1/8 are calculated, respectively, as shown in Figure S27 and Table S2. Compared with the high coverage of H and OH on Ni and NiO, the lower coverages will lead to a stronger H and OH adsorption interaction, hinting that too crowded H or OH on Ni and NiO surfaces have the repulsion interaction with each other to make them hard to adsorb on the surface. Nevertheless, for the different coverages, both H and OH bind weakly to the pure NiO surface, while metallic Ni surface shows a preference for binding H. The adsorption energies of 1/4 coverages are shown in Figure 4g.

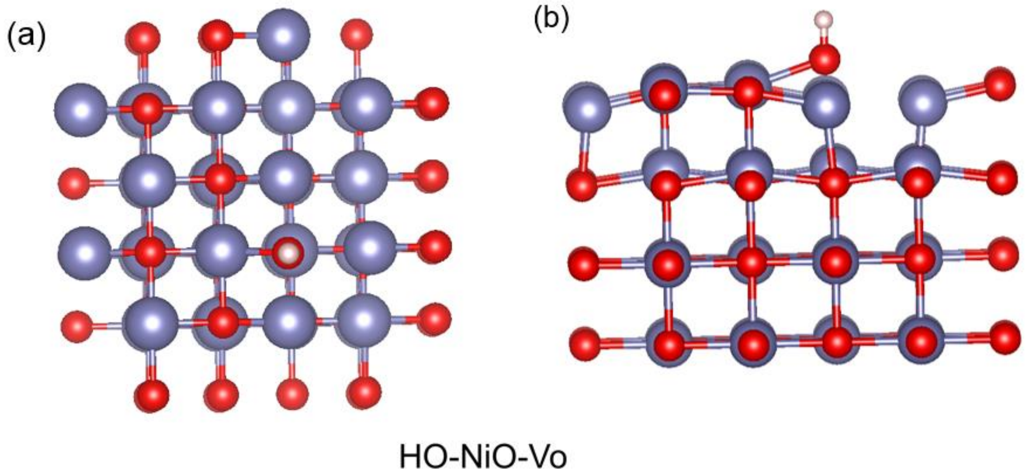

Figure S28. The optimal absorption site of O vacancies coupled NiO for OH.

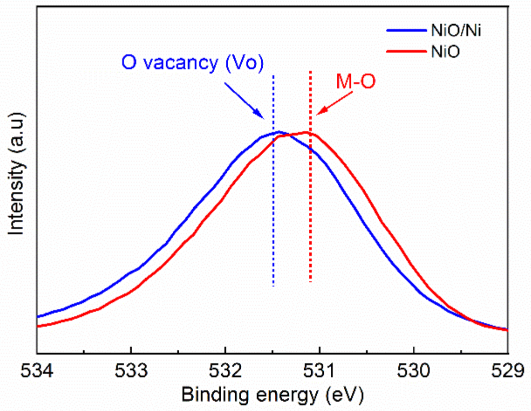

Figure S29. O 1*s* spectra of NiO/Ni and NiO.

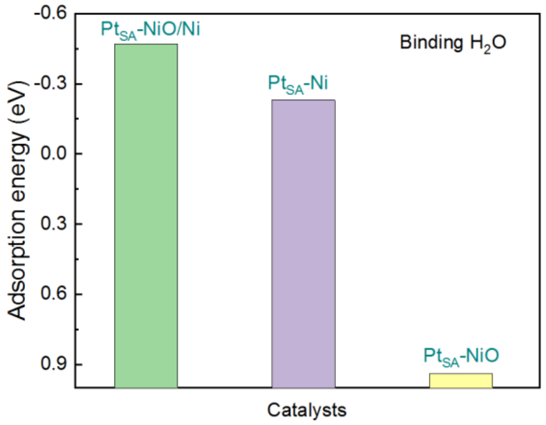

Figure S30. Calculated H_2O adsorption energies of Pt$_{SA}$-NiO/Ni, Pt$_{SA}$-NiO, and Pt$_{SA}$-Ni catalysts

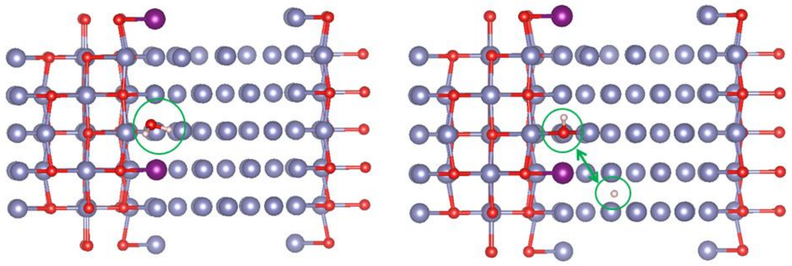

Figure S31. The optimal absorption site of Pt_{SA}-NiO/Ni catalyst for the dissociation of H_2O into OH and H.

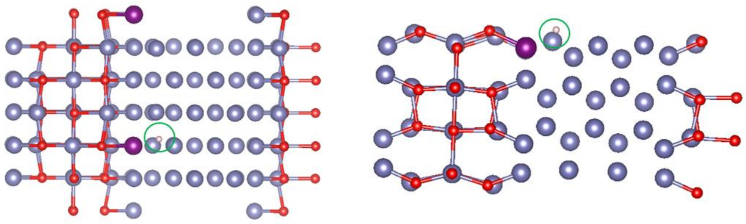

Figure S32. The optimal absorption site of Pt_{SA}-NiO/Ni catalyst for the dissociated proton.

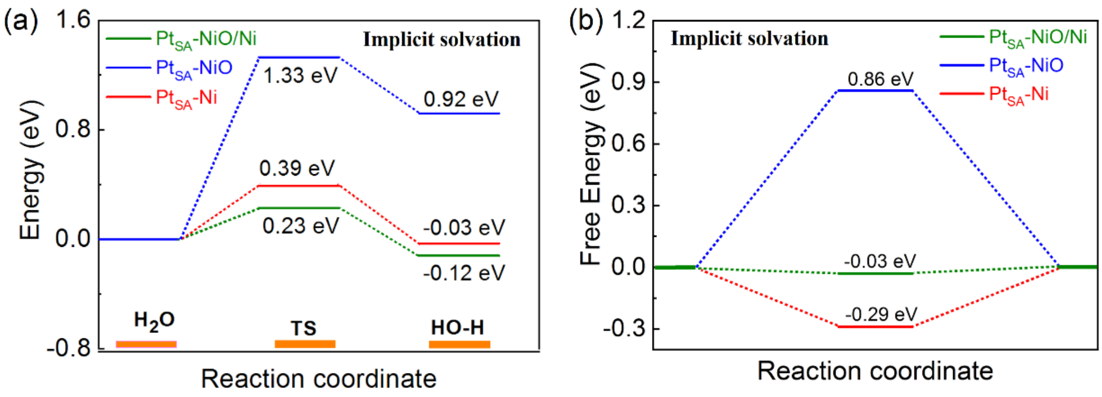

Figure S33. Calculated energy barriers of (a) water dissociation kinetic and (b) adsorption free energies of H* on the surface of the Pt$_{SA}$-NiO/Ni, Pt$_{SA}$-NiO, and Pt$_{SA}$-Ni catalysts by considering the effects of implicit solvation, respectively.

The effects of implicit solvation were considered by using VASPsol software,[4] and the energy barrier of water desorption and the adsorption free energies of H* for Pt$_{SA}$-NiO/Ni, Pt$_{SA}$-NiO, and Pt$_{SA}$-Ni systems were shown in Figure S33a-b. Under the effect of implicit solvation, NiO/Ni coupled single-atom Pt catalyst demonstrates the minimum energy barriers (0.23 eV) for the dissociation of H_2O into OH and H than that of NiO coupled single-atom Pt and Ni coupled single-atom Pt catalyst (Figure S33a), confirming the critical role of surface-exposed NiO/Ni interfaces for the H_2O dissociation of Volmer step. Moreover, compared with Pt$_{SA}$-NiO and Pt$_{SA}$-Ni systems, the NiO/Ni supported single-atom Pt sites at the NiO/Ni interfaces also show near-zero H binding energy (-0.03 eV, Figure S33b), which is more favorable for the recombination of the dissociated proton (H*) and H_2 evolution.

For the effects of explicit solvation shown in Figure 4h-i, we employed the Ab Initio Cluster-Continuum Model[5,6] to reduce the requirement of the number of configurations and water molecules, which strategy converges solvation energies by surrounding an explicit water shell with the implicit solvent outside. The explicit water molecules were set around the catalytic active site of the model surface, and 100 configurations were obtained by the LAMMPS molecular dynamics software.[7] After testing the explicit water molecules with the different numbers of 5, 14, 25, 35, 75, and 103, we found that the 35 water molecules could ensure the energy converged. The explicit water molecules revised energy barriers of water dissociation and the

adsorption free energies of H* were shown in Figure 4h-i, respectively.

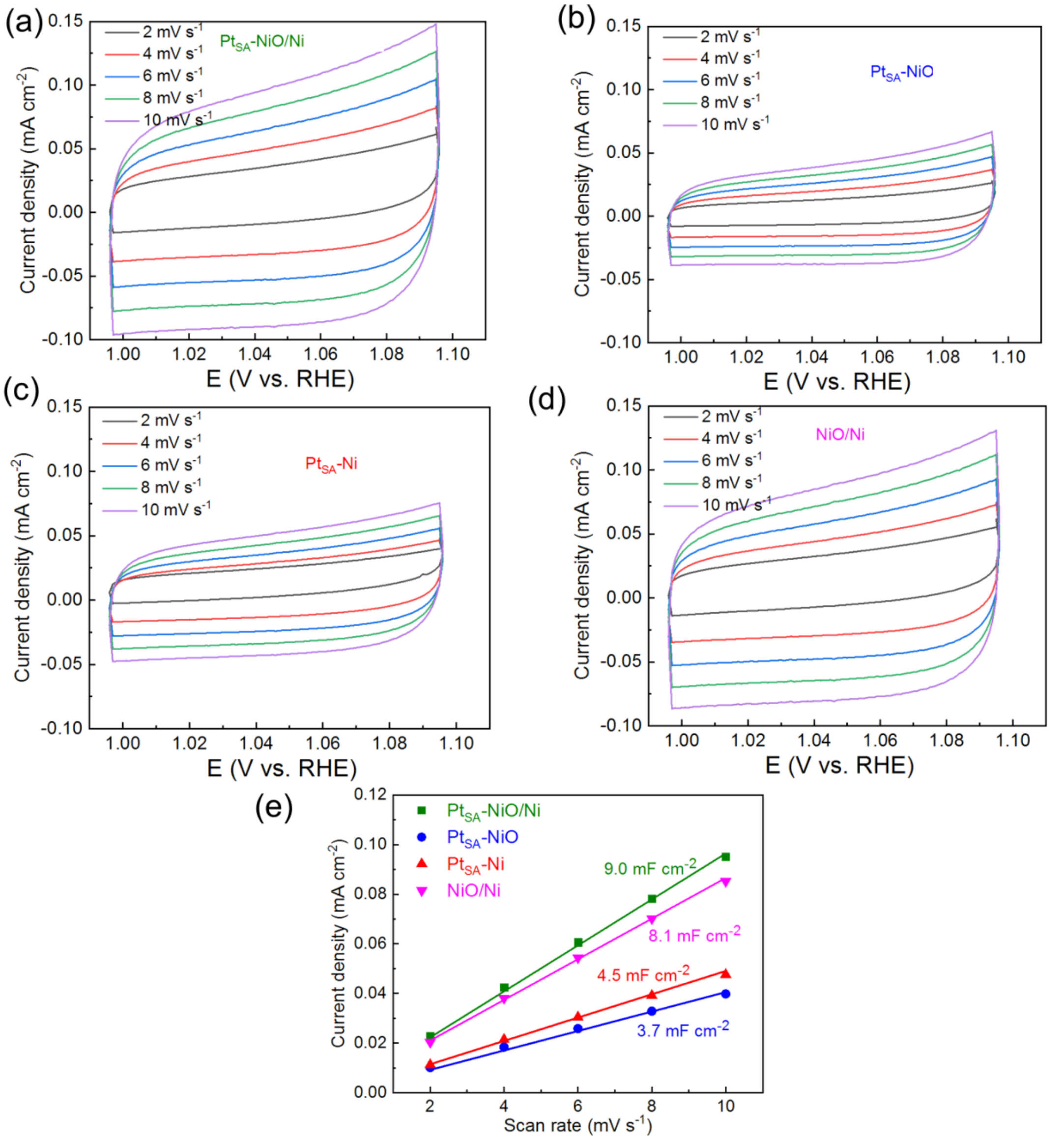

Figure S34. Cyclic voltammograms performed in 1 M KOH solution in the potential window without the participation of faradaic processes for (a) Pt$_{SA}$-NiO/Ni, (b) Pt$_{SA}$-NiO, (c) Pt$_{SA}$-Ni, and (d) NiO/Ni. (e) The corresponding scan rate dependence of the average currents.

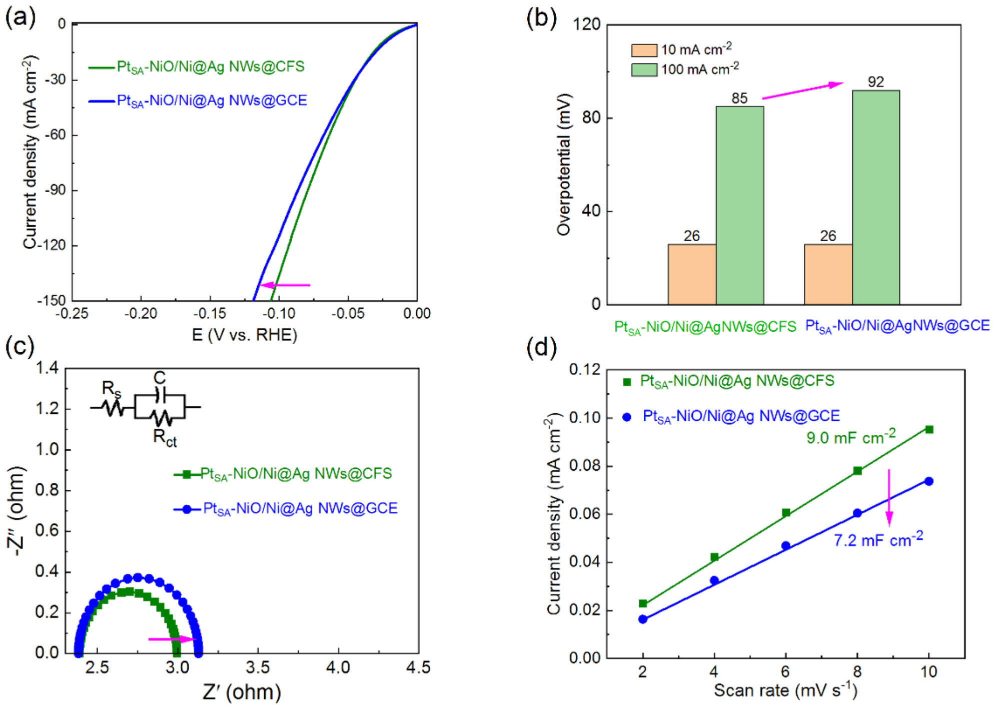

Figure S35. The alkaline HER performances of the fabricated Pt_{SA}-NiO/Ni@Ag NWs loaded on the traditional glass carbon electrode and cloth fabric substrate. (a) HER polarization curves. (b) The comparison of overpotentials required to achieve 10 and 100 mA cm^{-2} for various catalysts. (c) EIS Nyquist plots of the catalysts. (d) The corresponding scan rate dependence of the average currents.

Pt_{SA}-NiO/Ni supported by Ag NWs loaded glass carbon electrode (Pt_{SA}-NiO/Ni@AgNWs@GCE) was fabricated by coating catalyst ink on the current collector using Nafion as an adhesive followed by an electrochemical procedure. In detail, 5 mg Ag NWs, 50 µl Nafion (5 wt%), and 0.95 ml ethanol were mixed and then sonicated for 30 mins to form a dispersion solution. Then a part of the dispersion ink was dropped onto the glassy carbon electrode followed by drying at room temperature in a vacuum oven. The Ag NWs loading was determined to be ~0.47 mg cm^{-2}. Finally, Pt_{SA}-NiO/Ni@AgNWs@GCE was were prepared using the same procedure as the Pt_{SA}-NiO/Ni@Ag NWs loaded cloth fabric substrate (Pt_{SA}-NiO/Ni@AgNWs@CFS) except for choosing Ag NWs loaded glass carbon electrode instead of Ag NWs loaded cloth fabric substrate. It is necessary to note that for the Ag NWs loaded flexible cloth fabric, the polymer binders are unnecessary due to the strong affinity ability between Ag NWs and cloth fibers.

The alkaline HER performances of Pt_{SA}-NiO/Ni@AgNWs@GCE were

investigated under a standard three-electrode system. As shown in Figure S35a-b, the Pt_{SA}-NiO/Ni@AgNWs@GCE show a negligible difference in HER performances with Pt_{SA}-NiO/Ni@AgNWs@CFS when the response current density is lower than -50 mA cm^{-2}. However, when the current density is greater than -50 mA cm^{-2}, the HER performances of Pt_{SA}-NiO/Ni@AgNWs@GCE demonstrate inferior to that of Pt_{SA}-NiO/Ni@AgNWs@CFS. To get insight into the origin of the extraordinary HER performance of Pt_{SA}-NiO/Ni@AgNWs@CFS, the reaction kinetics of the fabricated electrodes were measured by EIS. As depicted in Figure S35c, the Pt_{SA}-NiO/Ni@AgNWs@CFS exhibits a much low R_{ct} value (0.61 Ω cm^{-2}) than Pt_{SA}-NiO/Ni@AgNWs@GCE (0.72 Ω cm^{-2}), indicating the smaller interfacial charge-transfer resistance for Pt_{SA}-NiO/Ni@AgNWs@CFS. Furthermore, the ECSA of the different electrodes was measured using the cyclic voltammetry technique to obtain C_{dl}. As shown in Figure S35d, the Pt_{SA}-NiO/Ni@AgNWs@CFS possessed the higher C_{dl} (9.0 mF cm^{-2}) than Pt_{SA}-NiO/Ni@AgNWs@GCE (7.2 mF cm^{-2}), suggesting more accessible active sites in Pt_{SA}-NiO/Ni@AgNWs@CFS.

From the above discussion, the preparation of the traditional glass carbon electrode-loaded catalysts usually involves coating catalyst fines on the current collectors using adhesives such as Nafion. However, the incorporation of these insulating adhesives will inevitably bury active sites and increases the dead volume and the contact resistance between the catalyst and the current collector,[8] causing the insufficient utilization of active sites and poor electron transferability as shown in Figure S35c-d. Thus, integrating catalysts and current collectors to form a seamlessly conductive electrode is necessary. The extraordinary HER performances of Pt_{SA}-NiO/Ni@AgNWs@CFS are attributed to the high conductivity, more accessible active sites, the favorable mass transfer, and facile hydrogen release originating from the binder-free electrode fabrication process, interconnected pores structure, and the super hydrophilic nature of the cloth fabric substrate supported catalyst as above discussion. Even so, the HER performances of Pt_{SA}-NiO/Ni@AgNWs@GCE are superior to most previously reported catalysts as shown in Figure 5h due to the high intrinsic HER activity of single-Pt anchored NiO/Ni hybrid system.

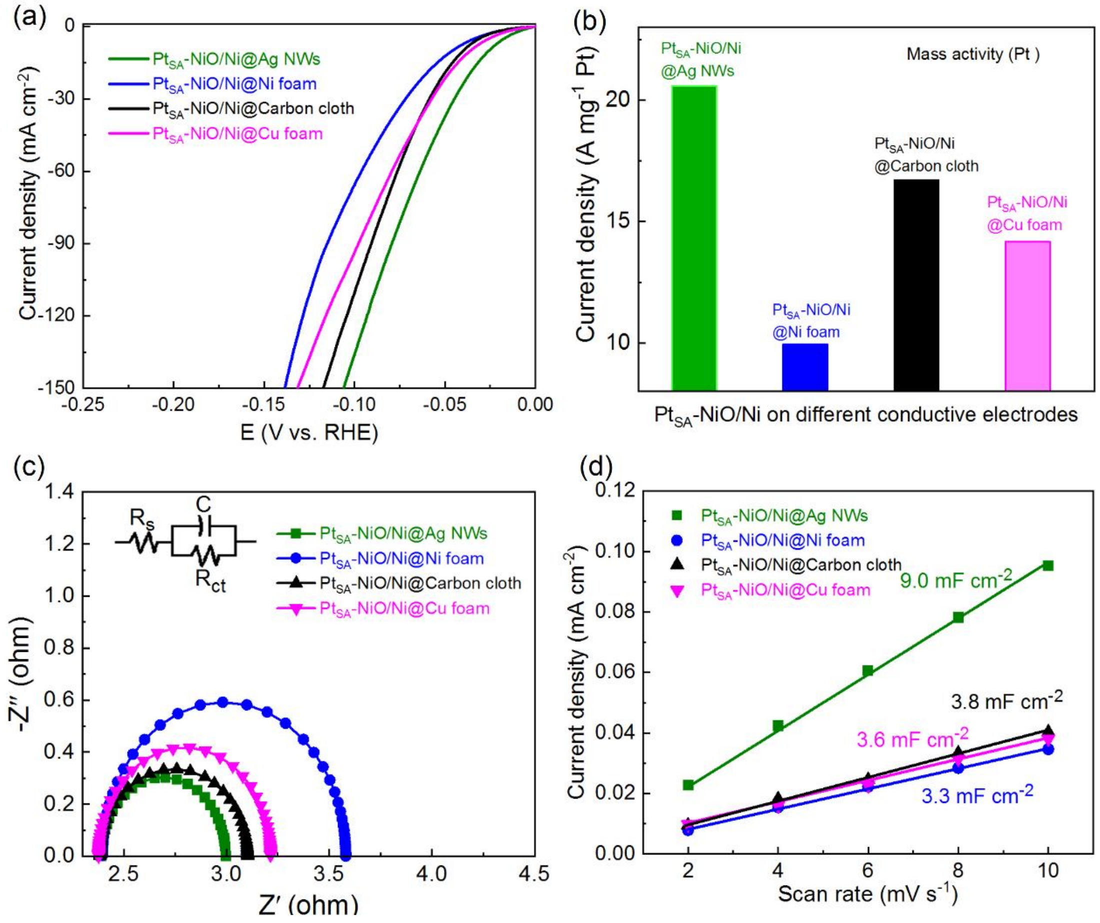

Figure S36. Electrocatalytic alkaline HER performances of the Pt_{SA}-NiO/Ni attached to the different conductive electrodes, including Ag NWs, Ni foam, carbon cloth, and Cu foam. (a) HER polarization curves, (b) the mass activity, (c) EIS Nyquist plots, and (d) C_{dl} of the catalysts.

Several catalytic systems with different conductive supporters were introduced as comparable groups. The Pt_{SA}-NiO/Ni@Ni foam, Pt_{SA}-NiO/Ni@Carbon cloth, and Pt_{SA}-NiO/Ni@Cu foam were prepared using the same procedure as the Pt_{SA}-NiO/Ni@Ag NWs except for choosing Ni foam, carbon cloth, and Cu foam as the conductive supporter, respectively. As shown in Figure S36a-b, the response current density of Pt_{SA}-NiO/Ni@Ag NWs was higher than other control groups at the same overpotential in the polarization curve, suggesting a superior electrocatalytic HER activity. The Pt_{SA}-NiO/Ni@Ag NWs catalytic system could deliver a current density of 10 mA cm^{-2} at an overpotential of 27 mV, which is lower than that of the Pt_{SA}-NiO/Ni@Ni foam (46 mV), Pt_{SA}-NiO/Ni@Carbon cloth (40 mV), and Pt_{SA}-NiO/Ni@Cu foam (36 mV). Moreover, the mass activity of Pt_{SA}-NiO/Ni@Ag NWs normalized to the loaded Pt mass at an overpotential of 100 mV is 20.6 A mg^{-1} (Figure S36b), which is about 2.1, 1.2, and 1.5

times greater than that of Pt_{SA}-NiO/Ni@Ni foam (9.9 A mg^{-1}), Pt_{SA}-NiO/Ni@Carbon cloth (16.7 A mg^{-1}) and Pt_{SA}-NiO/Ni@Cu foam (14.2 A mg^{-1}), respectively, suggesting that introducing Ag NWs into Pt_{SA}-NiO/Ni can extremely maximize the alkaline HER activity of Pt-based catalysts.

To get insight into the origin of the extraordinary HER performance of Ag NWs supported Pt_{SA}-NiO/Ni, the HER reaction kinetics of the fabricated Pt-based catalysts were measured by EIS. As depicted in Figure S36c, Pt_{SA}-NiO/Ni@Ag NWs electrode exhibits a much lower R_{ct} value (0.61 Ω cm^{-2}) than that of Pt_{SA}-NiO/Ni@Ni foam (1.19 Ω cm^{-2}), Pt_{SA}-NiO/Ni@Carbon cloth (0.72 Ω cm^{-2}), and Pt_{SA}-NiO/Ni@Cu foam (0.84 Ω cm^{-2}), indicating Pt_{SA}-NiO/Ni@Ag NWs electrode holds a smaller interfacial charge-transfer resistance than the others. This is originated from the higher electronic conductibility of Ag NWs in the Pt_{SA}-NiO/Ni@Ag NWs electrode comparing with traditional Ni foam, carbon cloth, and Cu foam substrate,[9] which greatly accelerate interfacial charge transfer and mass transfer.[10]

Furthermore, ECSA of the Pt_{SA}-NiO/Ni attached to the different conductive electrodes was measured using the cyclic voltammetry technique to obtain the C_{dl}, which was linearly proportional to the ECSA. As shown in Figure S36d, Pt_{SA}-NiO/Ni@Ag NWs electrode possessed a considerably high C_{dl} (9.0 mF cm^{-2}) than Pt_{SA}-NiO/Ni@Ni foam (3.3 mF cm^{-2}), Pt_{SA}-NiO/Ni@Carbon cloth (3.8 mF cm^{-2}), and Pt_{SA}-NiO/Ni@Cu foam (3.6 mF cm^{-2}), suggesting much more accessible active sites in Pt_{SA}-NiO/Ni@Ag NWs, which contributes to the superior HER performance of Pt_{SA}-NiO/Ni@Ag NWs over Pt_{SA}-NiO/Ni@Ni foam, Pt_{SA}-NiO/Ni@Carbon cloth, and Pt_{SA}-NiO/Ni@Cu foam. From the above discussion, the Ag NWs possess outstanding features in the design of electrocatalyst for the highly efficient hydrogen evolution due to its unique nanostructure feature and high electron conductivity.

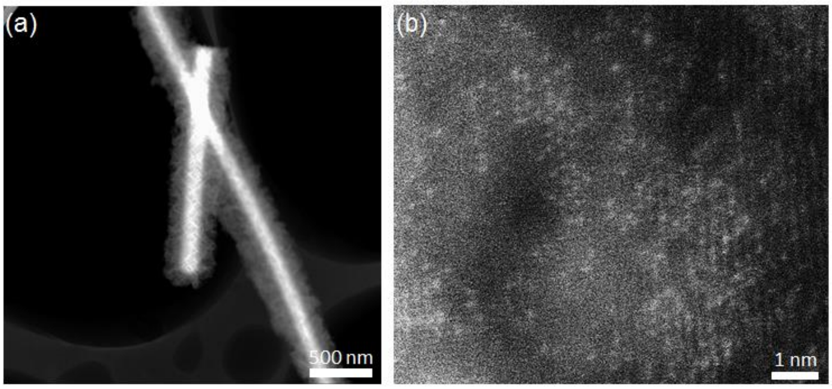

Figure S37. HAADF-STEM images of Pt_{SA}-NiO/Ni after the stability test.

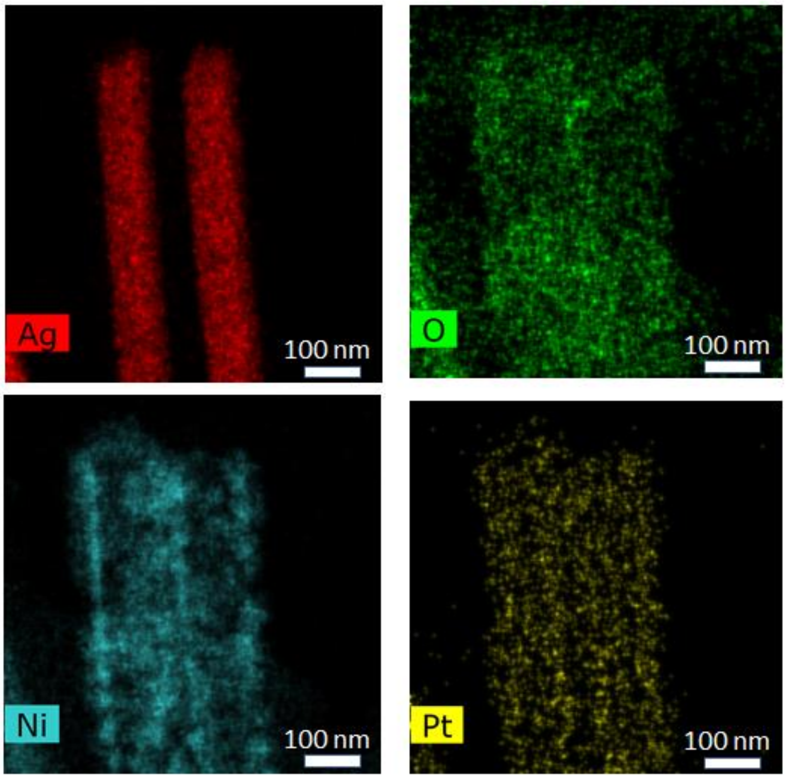

Figure S38. Elemental mapping of Pt_{SA}-NiO/Ni after the stability test.

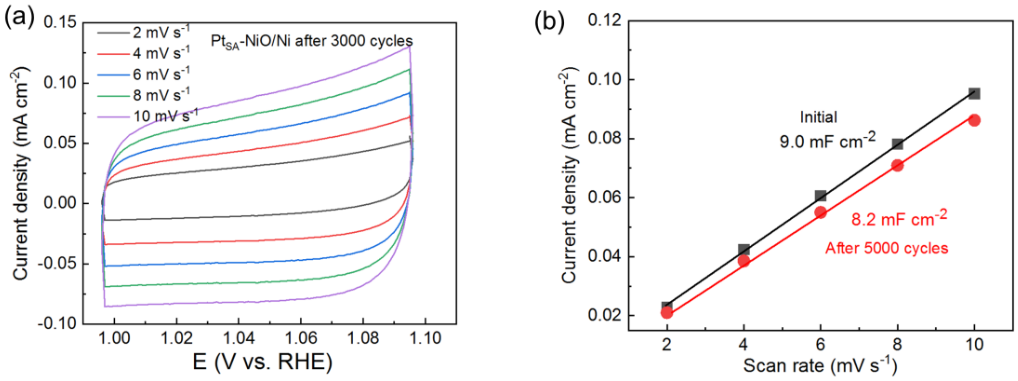

Figure S39. The double-layer capacitance measurement of Pt_{SA}-NiO/Ni after the stability test.

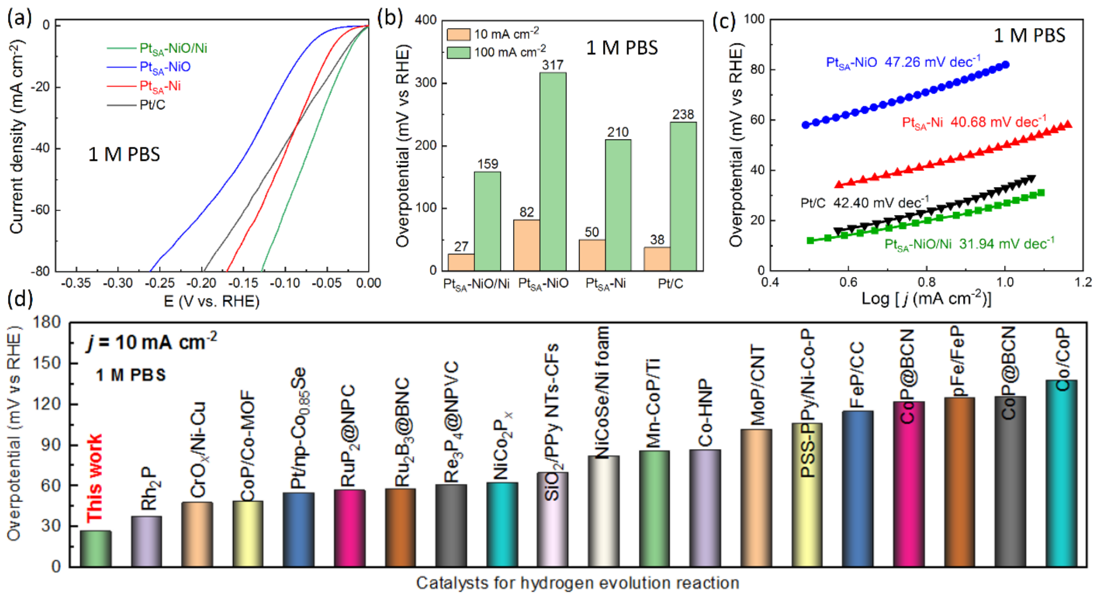

Figure S40. Electrocatalytic HER performances of the catalysts in 1 M PBS neutral electrolyte. (a) HER polarization curves of Pt$_{SA}$-NiO/Ni, Pt$_{SA}$-NiO, Pt$_{SA}$-Ni, and Pt/C. (b) The comparison of overpotentials required to achieve 10 and 100 mA cm^{-2} for various catalysts. (c) Corresponding Tafel slope originated from LSV curves. (d) Comparison of the neutral HER activity for Pt$_{SA}$-NiO/Ni with reported catalysts, originating from Table S4.

Table S1. FT-EXAFS fitting results of Pt_{SA}-NiO/Ni, Pt_{SA}-NiO and Pt_{SA}-Ni, and Pt foil was given as a reference.

	Shell	*CN*	*R*(Å)	σ^2	ΔE_0	*R* factor
Pt foil	Pt-Pt	12	2.76±0.01	0.004	10.4±0.6	0.0017
Pt_{SA}-NiO/Ni	Pt-Ni	5.87±0.5	2.60±0.01	0.012	9.2±2.0	0.0095
	Pt-O	1.31±0.2	1.99±0.02	0.001		
	Pt-Cl	1.91±0.5	3.10±0.02	0.004		
Pt_{SA}-NiO	Pt-Ni	2.14±0.2	2.70±0.01	0.005	18.6±1.8	0.0099
	Pt-O	2.41±0.2	2.07±0.02	0.009		
	Pt-Cl	1.62±0.3	3.20±0.02	0.004		
Pt_{SA}-Ni	Pt-Ni	4.97±0.5	2.63±0.01	0.010	16.9±2.4	0.0054
	Pt-Cl	1.40±0.2	2.31±0.01	0.002		

CN: coordination numbers of identical atoms; *R*: interatomic distance; σ^2: Debye-Waller factors; ΔE_0: energy shift. *R* factor: goodness of fit. S_0^2 was set to 0.74, according to the experimental EXAFS fit of Pt foil reference by fixing *CN* as the known crystallographic value.

Table S2. H and OH adsorption energies on Ni (100) and NiO (100) surface with the different coverages of 1/1, 3/4, 1/2, 1/4, and 1/8, respectively. The unit is eV.

	1/1	3/4	1/2	1/4	1/8
Ni_H	-0.5253	-0.5219	-0.5186	-0.5177	-
Ni_OH	0.2252	0.0406	-0.2496	-0.1852	-
NiO_H	0.4506	0.2162	0.3986	-0.1123	-0.1221
NiO_OH	1.0381	0.8177	1.0001	0.7182	0.8234

Table S3. Comparison of HER performances for Ag NWs supported Pt_{SA}-NiO/Ni with the reported HER catalysts in alkaline electrolytes.

	Electrocatalysts	Electrolyte	Overpotential at 10 mA cm^{-2} (mV)	Tafel slop (mV dec^{-1})	Reference
**1**	**Pt_{SA}-NiO/Ni**	**1.0 M KOH**	**26**	**27**	**This work**
2	Pt_{SA}-C_1N_1	1.0 M KOH	46	36.8	Nat. Commun., 2020, 11:1029.
3	Ru-MoS_2/CC	1.0 M KOH	41	114	Appl. Catal., B, 2019, 249, 91.
4	Pt_{SA}-$Co(OH)_2$	1.0 M KOH	29	35	Energy Environ. Sci., 2020, 13:3082.
5	RuCo@N-C	1.0 M KOH	28	31	Nat. Commun., 2017, 8, 14969.
6	Ru-MoO_2	1.0 M KOH	29	44	J. Mater. Chem. A, 2017, 5, 5475-5485.
7	PtNi NWs/C	1.0 M KOH	40	-	Angew. Chem.Int. Ed., 2016, 55, 12859.
8	IrCo@N-C	1.0 M KOH	45	80	Adv. Mater., 2018, 30,1705324.
9	Hexagonal-PtNi	1.0 M KOH	65	74	Nat. Commun., 2017, 8, 15131.
10	Ru/C_3N_4/C	0.1 M KOH	79	-	J. Am. Chem. Soc., 2016, 138, 16174.
11	$Cu_{2-x}S$@Ru	1.0 M KOH	82	48	Small, 2017, 13, 1700052.
12	PtNi-O/C	0.1 M KOH	40	79	J. Am. Chem. Soc., 2018, 140, 9046.
13	Pt-Ni ASs	1.0 M KOH	28	27	Adv. Mater., 2018,30, 1801741.
14	$Pt_{3.6}$Ni-S NWs	0.1 M KOH	38	-	Angew Chem. Int. Ed., 2018, 130,11852.
15	Rh_2P	1.0 M KOH	30	-	Adv. Energy Mater., 2018, 8,1703489.
16	hcp excavated PtNi	0.1 M KOH	38	42	Inorg. Chem. Front., 2018,5, 1365.
17	$Ni(OH)_2$-PtO_2 NS/Ti	0.1 M KOH	44	89	J. Mater. Chem. A, 2018, 6, 1967.
18	Pt_3Ni_2 NWs-S/C	0.1 M KOH	45	-	Nat. Commun., 2017, 8, 14580.
19	$Mo_2TiC_2T_x$-Pt_{SA}	0.5 M H_2SO_4	30	30	Nat. Catal., 2018, 1, 985.
20	Pt_{SA}/OLC	0.5 M H_2SO_4	38	36	Nat. Energy, 2019, 4, 512.
21	Pt-MWCNTs	0.5 M H_2SO_4	44	30	Nano Energy, 2019, 63, 1038.

22	Pt-MoS_2	0.1 M H_2SO_4	60	96	Energy Environ. Sci., 2015, 8, 1594.
23	er-WS_2-Pt	0.5 M H_2SO_4	40	27	Adv. Mater., 2017, 1704779.
24	Pt@PCM	0.5 M H_2SO_4	105	63	Sci. Adv., 2018, 4, 6657.
25	ALD50 Pt/NGNs	0.5 M H_2SO_4	39	29	Nat. Commun., 2016, 7, 13638.
26	Pt_{SA}/m-WO_{3-x}	0.5 M H_2SO_4	38	45	Angew. Chem., Int. Ed., 2019, 131, 16184.
27	Pt_{SA}/S-C	0.5 M H_2SO_4	53	46	Nat. Communicat., 2019, 10, 1.
28	Ru/N-C NWs	0.1 M H_2SO_4	47	14	Nat. Communicat., 2019, 10, 1.
29	Pd-Graphdiyne	0.5 M H_2SO_4	55	47	iScience, 2019, 11, 31.
30	PtN_x/TiO_2	0.5 M H_2SO_4	67	34	Nano Energy, 2020, 73, 104739
31	PtML/Au NF/Ni foam	0.5 M H_2SO_4	60	53	Sci. Adv., 2015, 1, e1400268.
32	Rh/SiNW	0.5 M H_2SO_4	85	24	Nat. Commun., 2016, 7, 12272.
33	Pt@PM	0.5 M H_2SO_4	106	65	Sci. Adv., 2018, 4, eaao6657.
34	RuP_2@NPC	0.5 M H_2SO_4	38	38	Angew. Chem., Int. Ed., 2017, 56, 11559.
35	NiO-Ni/CNT	1.0 M KOH	86	82	Nat. Commun., 2014, 5, 4695.
36	Ni NP/Ni-NC	1.0 M KOH	147	114	Energy Environ. Sci., 2019, 12, 149.
37	Ni/NiP	1.0 M KOH	130	58	Adv. Funct. Mater., 2016, 26, 3314.
38	CoP/NCNHP	1.0 M KOH	115	66	J. Am. Chem. Soc., 2018, 140, 2610.
39	Co_2P	1.0 M KOH	160	61	Adv. Mater., 2017, 29, 1606980.
40	NiCoN/C nanocages	1.0 M KOH	103	-	Adv. Mater., 2019, 31, 1805541.
41	Co doped β-Mo_2C	1.0 M KOH	141	62	Adv. Funct. Mater., 2020, 30, 2000561.
42	NiP_2 NS/CC	1.0 M KOH	102	64	Nanoscale, 2014, 6, 13440.

Table S4. Comparison of HER performances for Ag NWs supported Pt_{SA}-NiO/Ni with the reported HER catalysts in neutral electrolytes.

	Electrocatalysts	Electrolyte	Overpotential at 10 mA cm^{-2} (mV)	Tafel slop (mV dec^{-1})	Reference
**1**	**Pt_{SA}-NiO/Ni**	**1 M PBS**	**27**	**32**	**This work**
2	RuP_2@NPC	1 M PBS	57	87	Angew. Chem., 2017, 129, 11717.
3	Pt/np-$Co_{0.85}$Se	1 M PBS	55	35	Nat. Commun., 2019, 10, 1743.
4	Rh_2P	1 M PBS	38	46	Adv. Energy. Mater., 2018, 8, 1703489.
5	Co-HNP	1 M PBS	87	42	Angew. Chem. Int. Ed., 2016, 55, 6725.
6	CoP/Co-MOF	1 M PBS	49	63	Angew. Chem. Int. Ed., 2019, 58, 4679.
7	$NiCo_2P_x$	1 M PBS	63	63	Adv. Mater., 2017, 29, 1605502.
8	Ru_2B_3@BNC	1 M PBS	58	70	Nano Energy, 2020, 75, 104881.
9	SiO_2/PPy NTs-CFs	1 M PBS	70	100	Angew. Chem. Int. Ed., 2017, 56, 8120.
10	PSS-PPy/Ni-Co-P	1 M PBS	106	81	Chem. Eng. J., 2021, 1, 129232.
11	CoP@BCN-1	1 M PBS	122	59	Adv. Energy Mater., 2017, 7, 1601671.
12	Mn-CoP/Ti	1 M PBS	86	82	ACS Catal., 2017, 7, 98.
13	Co/CoP	1 M PBS	138	72	Adv. Energy Mater., 2017, 7, 1602355.
14	NiCoSe/Ni foam	1 M PBS	82	78	Adv. Mater., 2017, 29, 1606521.
15	MoP/CNT	1 M PBS	102	109	Adv. Funct. Mater., 2018, 28, 1706523.
16	pFe/FeP	1 M PBS	125	66	Chem. Eng. J., 2021, 408, 127330.
17	FeP/CC	1 M PBS	115	70	ACS Appl. Mater. Interfaces, 2014, 6, 20579.
18	Re_3P_4@NPVC	1 M PBS	61	77	Appl. Catal. B. Environ., 2019, 256, 117851.
19	CoP@BCN	1 M PBS	126	104	Adv. Energy Mater., 2017, 7, 1601671.
20	CrO_x/Ni-Cu	1 M PBS	48	64	Nat. Energy, 2019, 4, 107.

Supplementary Note 1. Turnover frequency (TOF) calculation of the Pt-based catalysts.

The TOF per Pt site of the fabricated Pt-based catalysts in this work was calculated by using the formula:

$$\text{TOF } (H_2/s) = \frac{\text{\# total hydrogen turnover per geometric area}}{\text{\# active sites per geometric area}} \quad (1)$$

The total number of hydrogen turnovers could be obtained by the current density as the following formula:

total hydrogen turnover:

$$= \left(|j|\frac{\text{mA}}{\text{cm}^2}\right)\left(\frac{1\ \text{C/s}}{1000\ \text{mA}}\right)\left(\frac{1\ \text{mol e}^-}{96485.3\ \text{C}}\right)\left(\frac{1\ \text{mol}}{2\ \text{mol e}^-}\right)\left(\frac{6.022\times10^{23}\ \text{molecules } H_2}{1\ \text{mol } H_2}\right) \quad (2)$$

$$= 3.12\times 10^{15}\ \frac{H_2/s}{\text{cm}^2}\ \frac{\text{mA}}{\text{cm}^2} \quad (3)$$

The number of catalytic active sites in the Pt-based catalysts was calculated according to the mass loading on the electrode, the Pt contents, and the Pt atomic weight with a hypothesis that each Pt atom accounts for one catalytic active site:

Pt active site:

$$=\left(\frac{\text{catalyst loading per geometric area } (x\ \text{g/cm}^2)\times\text{Pt wt\%}}{\text{Pt } M\text{w (g/mol)}}\right)\times\left(\frac{6.022\times10^{23}\text{Pt atoms}}{1\ \text{mol Pt}}\right) \quad (4)$$

Hence, the Pt active site in Pt_{SA}-NiO/Ni can be calculated to be:

$$= \left(\frac{0.0066\times10^{-3}}{195.08\ \text{g/mol}}\right)\times\left(\frac{6.022\times10^{23}\text{Pt atoms}}{1\ \text{mol Pt}}\right) \quad (5)$$

$$= 2.03\times 10^{16} \quad (6)$$

So, the TOFs of per Pt site in Pt_{SA}-NiO/Ni can be calculated to be:

$$= \frac{3.12\times10^{15}}{2.03\times10^{16}}\times|j| \quad (7)$$

$$= 0.18\times|j| \quad (8)$$

Supplementary References

1 Kothari, V. Polyesters and polyamides. *Elsevier*, 419-440 (2008).

2 Lin, C. Polyesters and polyamides. *Elsevier*, 62-96 (2008).

3 Wu, L. *et al.* Boosting vanadium flow battery performance by Nitrogen-doped carbon nanospheres electrocatalyst. *Nano Energy* **28**, 19-28 (2016).

4 Mathew, K. *et al.* Implicit solvation model for density-functional study of nanocrystal surfaces and reaction pathways. *J. Chem. Phys.* **140**, 084106 (2014).

5 Pliego, J. R. *et al.* The cluster-continuum model for the calculation of the solvation free energy of ionic species. *J. Phys. Chem. A* **105**, 7241-7247 (2001).

6 Patel, A. M. *et al.* Theoretical approaches to describing the oxygen reduction reaction activity of single-atom catalysts. *J. Phys. Chem. C* **122**, 29307-29318 (2018).

7 Plimpton, S. Fast parallel algorithms for short-range molecular dynamics. *J. Comput. Phys.* **117**, 1-19 (1995).

8 Zhang, Y. *et al.* Ultrafine metal nanoparticles/N-doped porous carbon hybrids coated on carbon fibers as flexible and binder-free water splitting catalysts. *Adv. Energy Mater.* **7**, 1700220 (2017).

9 Zhou, K. *et al.* A Setaria-inflorescence-structured catalyst based on nickel-cobalt wrapped silver nanowire conductive networks for highly efficient hydrogen evolution. *J. Mater. Chem. A* **7**, 26566-26573 (2019).

10 Sun, Q. *et al.* Synergistic nanotubular copper-doped nickel catalysts for hydrogen evolution reactions. *Small* **14**, 1704137 (2018).
